# Hypothyroidism as Prognostic Factor in Cutaneous Melanoma: A 1553‐Patient Single‐Center Study

**DOI:** 10.1002/cam4.71688

**Published:** 2026-03-17

**Authors:** Julie Klose, Filipa Almeida Oliveira, Charalampos Varnava, Philipp Wiebringhaus, Grit Sophie Herter‐Sprie, Hans‐Joachim Schulze, Tobias Hirsch, Sascha Wellenbrock, Maximilian Kueckelhaus

**Affiliations:** ^1^ Department of Plastic, Reconstructive and Aesthetic Surgery Hand Surgery, Fachklinik Hornheide Muenster Germany; ^2^ Department of Plastic and Reconstructive Surgery, Institute of Musculoskeletal Medicine University Hospital Muenster Muenster Germany; ^3^ Department of Plastic Surgery University Hospital Muenster Muenster Germany; ^4^ Department of Medical Oncology Fachklinik Hornheide Muenster Germany; ^5^ Faculty of Medicine University of Cologne Cologne Germany; ^6^ Department of Dermatology Fachklinik Hornheide Muenster Germany

**Keywords:** cutaneous melanoma, endocrine influence on cancer, hypothyroidism, prognostic factors

## Abstract

**Background:**

Cutaneous melanoma is a rising global health concern and identifying modifiable or associated risk factors remains essential for improving prognostic stratification. Hypothyroidism, affecting approximately 5.1% of the general population in Germany, has been associated with various malignancies. This study investigates the prevalence and potential prognostic implications of hypothyroidism in melanoma patients.

**Methods:**

We conducted a retrospective single‐centre study including 1553 patients with cutaneous melanoma (AJCC stages I–III). Patients were stratified based on thyroid function into hypothyroid and euthyroid groups. Data were collected via clinical records and standardized patient questionnaires.

**Results:**

Hypothyroidism was documented in 234 patients (15.1%), significantly higher than the national prevalence (5.1%, *p* < 0.001). Tumor site differed significantly between groups, with hypothyroid patients more frequently exhibiting melanomas on the lower extremities (*p* < 0.001). These patients also showed a non‐significant trend toward lower Breslow thickness (*p* = 0.09) and a significantly reduced number of metastatic lymph nodes (*p* = 0.009).

**Conclusions:**

Our findings suggest that hypothyroidism is markedly more prevalent among patients with cutaneous melanoma and may be associated with clinical features linked to improved prognosis. These observations warrant further investigation to clarify whether hypothyroidism itself, or its underlying immunologic and hormonal milieu, plays a protective role in melanoma progression.

## 1 Introduction

Cutaneous melanoma is a malignancy originating from melanocytic cells. With steadily rising incidence, 11,320 women and 12,240 men were diagnosed with melanoma in Germany in 2020, according to the Robert Koch Institute, with 2940 melanoma‐related deaths recorded that year [1]. In the United States, 76,830 individuals were diagnosed with invasive cutaneous melanoma in 2016 [2]. In Europe, incidence has risen from 3 to 4 cases per 100,000 in the 1970s to 10–15 cases per 100,000 in the early 2000s [3]. This rate of increase surpasses that of any other malignancy; however, mortality has not risen proportionally [4]. Although melanoma constitutes only approximately 3% of all skin cancers, it accounts for nearly 65% of all skin cancer‐related deaths [5].

The biological progression of melanoma is classically divided into two stages: an initial radial growth phase, in which tumor cells proliferate laterally within the basal epidermis, and a subsequent vertical growth phase, characterized by basement membrane penetration and potential for metastasis [6, 7]. According to the tumor progression model proposed by Clark et al., metastasis represents the final evolutionary step in melanoma [7]. During vertical growth, melanoma cells secrete angiogenic factors such as vascular endothelial growth factor (VEGF) [8], promoting lymphatic and hematogenous spread—a process associated with poorer prognosis in the setting of increased vascular density [9].

Well‐established prognostic factors for melanoma progression include tumor thickness, anatomical location, and patient gender [10, 11]. Approximately half of all metastasizing melanomas initially spread to regional lymph nodes, while the remainder manifest as satellite/in‐transit metastases (21.7%) or distant metastases (28.1%) [12].

Thyroid hormone metabolism, particularly hypothyroidism, has already been linked to cancer biology in several tumor entities, including breast and renal cell carcinoma [13, 14, 15]. However, its role in the context of cutaneous melanoma remains poorly defined. Given this background, we conducted a large retrospective analysis of 1553 melanoma patients (AJCC stages I–III), comparing individuals with and without hypothyroidism to explore possible correlations between thyroid dysfunction and prognostic tumor features.

The results aim to contribute to a deeper understanding of endocrine‐immune interactions in oncologic progression and support further prospective investigation.

## 2 Methods

This study was approved by the local institutional review board of the Medical Council Westfalen‐Lippe (ref. 2020‐822‐f‐S). This study was a retrospective analysis of institutional data. A waiver of written informed consent was approved by the local ethics committee due to the retrospective nature of the study.

Patient data was collected retrospectively from the hospital's electronic patient records.

All melanoma patients aged 18 years and older who underwent sentinel lymph node dissection (SLND)—and complete axillary or inguinal dissection if deemed necessary—between January 2013 and September 2018 were recorded.

Hypothyroidism was defined exclusively as pharmacologically treated hypothyroidism, based on a documented history of thyroid hormone replacement therapy (e.g., L‐thyroxine). Only patients actively taking such medication were classified as hypothyroid. Subclinical hypothyroidism or untreated thyroid dysfunction was not considered for this analysis, and biochemical confirmation (TSH, free T3, free T4) was not routinely available.

Data were collected from the patients' first outpatient appointment for pre‐operative planning until their last follow‐up appointment institution. Data assignment was pseudonymized via the consecutive patient number. Collected data included surgery documentation, patient questionnaires, results of dermatological and histological examinations, and scanned documents from external hospitals submitted by the patients.

### 2.1 Statistical Analysis

To evaluate the significance whether the proportion of hypothyroid patients in this study population was higher than in the normal population, the binomial test was used. For correlations of categorical characteristics, the Fisher test was performed. For continuous characteristics, the individual points were described using mean, standard deviation, median, minimum, and maximum. The odds ratio was used to express any dependencies. In the absence of a normal distribution of continuous characteristics, the Mann–Whitney U test was used to test the similarity of two groups with continuous target variables.

Logistic regression analysis was performed to determine the influence of a continuous variable (TSH‐value) on a binary target variable (ulceration, lymph node metastases at initial diagnosis, capsular rupture).

The Spearman correlation coefficient correlates two continuous non‐normally distributed target variables; here, the TSH‐value and the number of affected lymph nodes.

For all statistical analyses, a *p* value < 0.05 was assumed to be statistically significant.

## 3 Results

### 3.1 Study Population

During the study period, 876 procedures on the axilla, as well as 677 procedures on the groin region on patients with cutaneous melanoma were performed. The patient population described consisted of a total of 1553 patients. 871 of these patients received their follow‐up care at the authors' institution. 757 (48.7%) patients were male and 796 (51.3%) were female. The age of the patients ranged from 21 to 95 years with a mean age of 59.02 years (standard deviation 15.23 years). There were 410 (33.9%) patients in tumor stage I, 333 (27.5%) in stage II, followed by 466 (38.5%) in tumor stage III at the time of surgery.

Of the 234 patients with cutaneous melanoma who simultaneously reported hypothyroidism as a comorbidity, 52 (22.2%) were men and 182 (77.8%) were women. Additional baseline characteristics of the study population are summarized in Table 1.

**TABLE 1:** Characteristics of hypothyroid patients and control group.

	Hypothyroid patients	Others
Number	234	1319
Sex		
Male	52 (22.2%)	705 (53.4%)
Female	182 (77.8%)	614 (46.6%)
Age (in years)		
Average	60.7	58.7
Body mass index		
Average	27.4	27.3
Comorbidities		
Hypertension	112 (47.9%)	545 (41.3%)
Diabetes mellitus type II	26 (11.1%)	147 (11.1%)
Hypercholesterinemia	24 (10.3%)	109 (8.3%)
Cardiac arrhythmias	11 (4.7%)	91 (6.9%)

### 3.2 Melanoma Characteristics

The histologic type was further specified in 965 patients (62.14%). Already more than half of the patients with known tumor histology have superficial spreading melanoma (577 = 59.8%). 243 (25.2%) of the melanomas were nodular, 120 (12.4%) had acro lentiginous melanoma, and 26 (2.6%) melanomas were lentigo maligna melanoma.

305 (20.1%) melanomas were found to be ulcerated upon histologic examination (ulceration status remained unclear in 32 patients); mean tumor thickness was 2.84 mm (standard deviation 3.66 mm), with a total of tumors with Breslow tumor thickness of 0.04–75 mm.

### 3.3 Association Between Melanoma and Hypothyroidism

The prevalence of hypothyroidism in the German population is 5.1% [15]; whereas the prevalence in the study population affected by cutaneous melanoma is significantly higher with 15.1% (*p* < 0.001).

In terms of age distribution, both groups in our sample did not differ significantly: while the average age in patients with hypothyroidism was 60.70 years (standard deviation 14.72 years), the non‐hypothyroid patients in the studied collective averaged at 58.72 years (standard deviation 15.30 years). The *t*‐test yielded a *p* value of *p* = 0.062. There was only a trend toward earlier melanoma diagnosis in the non‐hypothyroid group.

Furthermore, comparing the absolute number of lymph nodes harboring melanoma cells, the mean was 0.4 (standard deviation: 0.8) in hypothyroid patients and 0.7 (standard deviation: 1.8) in non‐hypothyroid patients. With a *p* value of 0.052, this difference falls just short of statistical significance.

Overall, 293 (20%) melanomas were ulcerated, with 41 patients (18.4%) in hypothyroid patients compared to 252 (20.2%) in non‐hypothyroid patients. This finding is not statistically significant (*p* = 0,585).

Of the 522 patients with lymph node metastasis included, 107 (20.5%) showed a capsular rupture in the histopathological analysis, leading subsequently to an extracapsular spread. Among the 67 hypothyroid patients in this group, 14 (20.9%) had capsular rupture in at least one of the examined lymph nodes. This difference was not significant (*p* = 1.00).

There was a significant difference in primary tumor location as more melanomas in the non‐hypothyroid group were localized on the upper extremities or other areas drained by the axillary lymph nodes compared to the hypothyroid patients (*p* < 0.001) (see Table 2).

**TABLE 2:** Comparison of the primary tumor data between hypothyroid and non‐hypothyroid patients. They only differ in the area of their lymph drainage.

		Hypothyroid patients	Others	*p* value
Melanoma	SSM	77 (53.8%)	500 (60.8%)	Total: 0.154
	NMM	41 (28.7%)	202 (24.6%)	
	ALM	18 (12.6%)	102 (12.4%)	
	LMM	7 (4.9%)	18 (2.2%)	
Lymphovascular inflow area	Axillary	107 (45.7%)	769 (58.3%)	**Total: < 0.001**
	Inguinal	127 (54.3%)	550 (41.7%)	
Average tumor		2.44 mm	2.91 mm	0.168
Thickness Clark‐level	I		1 (0.1%)	Total: 0.73
	II	2 (1.1%)	25 (2.5%)	
	III	35 (20.0%)	218 (22.2%)	
	IV	121 (69.1%)	646 (65.7%)	
	V	17 (9.7%)	92 (9.4%)	
Ulceration		41 (18.4%)	252 (20.2%)	0.585
AJCC stadium	I	68 (35.7%)	342 (33.5%)	Total: 0.533
	II	55 (28.9%)	278 (27.2%)	
	III	67 (35.2%)	399 (39.2%)	

In the hypothyroid group, 107 out of 234 (45.7%) presented with a primary tumor in the axillary stromal area, whereas in the non‐hypothyroid group, 58.3% (769 out of 1319 patients) had a tumor in the upper extremity—or axillary stromal area (see Table 2).

### 3.4 Influence of the TSH Value

The thyroid‐stimulating hormone (TSH) level could be retrospectively determined in 114 cases; 34 patients of these were self‐reported to suffer from hypothyroidism, and the remaining 80 patients had no known thyroid dysfunction. As expected, TSH was slightly elevated in the hypothyroid group (2.87 mU/L, SD ± 2.79 mU/L), but not statistically significant (*p* = 0.453), compared to the control group (1.77, SD ± 0.88).

Logistic regression was used to compare TSH levels with binary characteristics (ulceration, lymph node metastases at initial diagnosis, capsular rupture). The Spearman correlation coefficient investigates possible correlations between increased TSH levels and the number of affected lymph nodes in patients with lymph node metastasis. No significant correlation was found in any of these assessments (see Table 3).

**TABLE 3:** Impact of the TSH level on different binary characteristics.

		*n*	Mean value	Standard deviation	*p* value OR (95%‐KI)
Distant metastases	No	80	2.2	1.7	0.761
	Yes	19	2	2.1	0.94 (0.65–1.37)
Lymph node‐metastases	No	24	1.8	0.9	0.117
	Yes	75	2.2	2	1.20 (0.96–1.50)
Capsule breakthrough	No	58	2.4	2.2	0.16
	Yes	17	1.7	1.2	0.77 (0.53–1.11)
Ulceration	No	67	2.2	1.9	0.654
	Yes	32	2	1.6	0.95 (0.74–1.21)

*Note:* No evidence was found suggesting that the TSH level has an impact on the presence of lymph node or distant metastases nor the capsule breakthrough or the ulceration status of the primary tumor.

## 4 Discussion

In this retrospective, monocentric study involving 1553 patients with cutaneous melanoma, we identified a significantly higher prevalence of hypothyroidism in melanoma patients compared to the general population, a lower rate of lymph node metastasis in hypothyroid patients, and a significant difference in primary tumor localization between the hypothyroid and euthyroid subgroups.

While the impact of hypothyroidism on tumor biology has been extensively studied in other malignancies such as renal cell and breast carcinomas [13, 14, 15, 16], literature on its role in cutaneous melanoma remains sparse. To date, only one comparable study, by Ellerhorst et al. [17], has investigated this association. In their analysis—like ours—hypothyroidism was not defined biochemically but based on patient self‐report, primarily inferred from medication use. Moreover, Ellerhorst's group did not stratify patients by melanoma subtype or tumor thickness, although these parameters are known to correlate with disease progression and prognosis [18, 19, 20, 21].

Consistent with our findings, neither Ellerhorst et al. nor our analysis observed significant differences in AJCC tumor staging between hypothyroid and euthyroid groups. They also reported a lower frequency of ulcerated melanomas among hypothyroid patients, although, as in our study, this did not reach statistical significance.

The potential biological relationship between thyroid dysfunction and melanoma is supported by the expression of TSH receptors in melanoma cells and nevi [22], and the autocrine production of thyrotropin‐releasing hormone (TRH) within melanoma tissue [23]. Beyond their known roles in physiological growth and metabolism, thyroid hormones—including T3, T4, and TSH—may modulate tumor development via mechanisms involving angiogenesis (e.g., integrin αVβ3) and downstream pathways such as PI3K and MAPK [13, 24]. These pathways may help explain the increased prevalence of hypothyroidism observed in our melanoma cohort. Whether hypothyroidism itself increases the risk for developing melanoma, however, remains unclear and warrants future prospective studies.

Our data suggest that hypothyroidism may be associated with more favorable melanoma characteristics. Hypothyroid patients were less likely to present with lymph node involvement—either sentinel or downstream nodes. Even when metastases were present, hypothyroid patients had significantly fewer affected nodes (*p* = 0.009) and showed a trend toward lower overall lymph node burden (*p* = 0.052). Given that micrometastatic involvement of the sentinel lymph node (SLN) is a key prognostic factor in melanoma [25, 26] and that SLN biopsy (SLNB) improves both disease‐specific and progression‐free survival [27], this observation may suggest a more indolent course in hypothyroid individuals.

In addition, we observed a distinct tumor localization pattern: patients with hypothyroidism were significantly more likely to have melanomas located on the lower extremities. Previous work has shown that melanomas of the trunk and the so‐called TANS regions (thorax, upper arm, neck, scalp) are associated with worse prognoses, particularly in stage II disease [28, 29, 30]. This may be influenced by differences in UV exposure patterns, ease of self‐detection, and regional lymphovascular anatomy [31, 32].

Although our data indicate that hypothyroid patients were less likely to present with ulcerated melanomas and had a lower lymph node burden, a trend toward earlier diagnosis in euthyroid patients was also noted. This may reflect differences in age, comorbidities, or health‐seeking behavior, as hypothyroidism is more prevalent in older individuals.

The hypothesis that hypothyroidism could be a risk factor for melanoma development is supported by data from Prinzi et al. [33], who demonstrated a significantly increased melanoma risk in younger women with benign thyroid disease. A reciprocal association between papillary thyroid carcinoma and melanoma has also been proposed [34], suggesting shared hormonal or immunological mechanisms. TRH and TSH receptors are expressed in melanocytes and melanoma cells [22, 23], and TSH has been shown to activate key signaling pathways such as cAMP and MAPK [35]. Experimental models, such as the zebrafish, support this interaction: elevated T4 levels reduce melanophore populations, whereas hypothyroidism increases them [36].

Furthermore, emerging cancer therapies, especially immune checkpoint inhibitors, are known to induce hypothyroidism as a treatment‐related adverse event [37]. This adds an additional layer of complexity to interpreting thyroid status in melanoma patients and raises the possibility that hypothyroidism may modulate immune or tumor‐related mechanisms beyond direct endocrine effects.

Taken together, our findings suggest a potentially protective role of hypothyroidism in the progression of cutaneous melanoma, though causality cannot be inferred. Further mechanistic studies exploring the endocrine‐immunologic interface in melanoma pathogenesis are warranted.

## 5 Limitations

The primary limitations of this study are its retrospective design and monocentric nature. Patient selection was limited to individuals referred to our plastic surgery department for lymph node biopsy and dissection, potentially excluding patients with thin melanomas or lesions in anatomically less accessible sites. This may have introduced selection and detection bias and may partially explain the higher prevalence of hypothyroidism observed compared with the general population.

Thyroid status was determined on clinical documentation and patient‐reported medication use of thyroid hormone replacement therapy; biochemical parameters (TSH, free T3, free T4) were not consistently available and therefore not used for classification. Consequently, only pharmacologically treated (overt) hypothyroidism was included, while subclinical or untreated thyroid dysfunction was excluded. This approach reduces heterogeneity and misclassification in a retrospective dataset but may underestimate the full spectrum of thyroid function alterations in melanoma patients.

Stage IV melanoma patients were deliberately excluded to avoid confounding by treatment‐induced thyroid dysfunction, particularly from immune checkpoint inhibitors and targeted therapies. Therefore, our findings cannot be extrapolated to metastatic disease treated with systemic therapy.

Although the overall cohort was large, the proportion of hypothyroid patients was relatively small, limiting the applicability of propensity score matching without substantial loss of statistical power. Accordingly, conventional comparative and regression‐based statistical methods were used, and the results should be interpreted as hypothesis‐generating.

Finally, information on prior thyroidectomy was incomplete for a subset of patients; however, available data indicate that only a small minority underwent thyroidectomy, predominantly for benign disease, and no cases were associated with known melanoma‐thyroid cancer syndromes.

Future prospective, multicentred studies should aim for data collection, with systematic biochemical thyroid assessment and inclusion of advanced‐stage disease warranted to further validate these findings.

## 6 Summary

Here, we identified that (a) a significantly increased prevalence of hypothyroidism can be observed in cutaneous melanoma patients with melanoma Stage I–III; (b) hypothyroidism in melanoma patients tends to be a prognostically favorable factor in our patient population; and (c) hypothyroidism is associated with prognostic factors that positively affect survival in patients with cutaneous melanoma.

Whether, conversely, patients with a hypothyroid metabolism have an increased risk of developing cutaneous melanoma remains to be proven. To further elucidate the influences of thyroid hormone metabolism on the pathogenesis of melanoma and to derive possible therapeutic approaches, the hormone regulation of melanoma cells must be understood in more detail.

## Author Contributions

**Julie Klose:** data curation (lead), formal analysis (lead), writing – original draft (equal). **Filipa Almeida Oliveira:** validation (supporting), writing – review and editing (supporting). **Charalampos Varnava:** formal analysis (supporting), investigation (supporting). **Philipp Wiebringhaus:** conceptualization (supporting). **Grit Sophie Herter‐Sprie:** project administration (supporting), writing – review and editing (supporting). **Hans‐Joachim Schulze:** formal analysis (supporting), validation (supporting). **Tobias Hirsch:** conceptualization (supporting), resources (supporting), validation (supporting). **Sascha Wellenbrock:** project administration (supporting), writing – review and editing (lead). **Maximilian Kueckelhaus:** conceptualization (lead), writing – original draft (lead).

## Funding

The authors have nothing to report.

## Ethics Statement

As all data analyzed in this study were fully anonymized and aggregated, ethical approval and informed consent were not required, in accordance with national legislation and institutional guidelines. The study was conducted in compliance with the Declaration of Helsinki and the European General Data Protection Regulation (GDPR).

## Conflicts of Interest

The authors declare no conflicts of interest.

## Data Availability

The data that support the findings of this study are available from the corresponding author upon reasonable request.
